# Does neighbourhood social capital aid in levelling the social gradient in the health and well-being of children and adolescents? A literature review

**DOI:** 10.1186/1471-2458-13-65

**Published:** 2013-01-23

**Authors:** Veerle Vyncke, Bart De Clercq, Veerle Stevens, Caroline Costongs, Giorgio Barbareschi, Stefán Hrafn Jónsson, Sara Darias Curvo, Vladimir Kebza, Candace Currie, Lea Maes

**Affiliations:** 1 Department of Public Health, Ghent University, De Pintelaan 185 blok A, 9000, Ghent, Belgium; 2 The Flemish Institute for Health Promotion and Disease Prevention, Gustave Schildknechtstraat 9, 1020, Brussels, Belgium; 3 EuroHealthNet, Rue de la loi 67, 1040, Brussels, Belgium; 4 University of Iceland and The Directorate of Health, Saemundargata 101, Reykjavik, Iceland; 5 Universidad de la Laguna, Pabellón de Gobierno, C/Molinos de Agua s/n, 38207, La, Laguna, Spain; 6 National Institute of Public Health, Srobarova 48, 10042, Prague, Czech Republic; 7 Child and Adolescent Health Research Unit (CAHRU), School of Medicine, University of St Andrews, Medical and Biological Sciences Building, North Haugh, St Andrews, Fife KY16 9TF, UK; 8 Present address: Department of General Practice and Primary Care, Ghent University, De Pintelaan 185, B-9000, Ghent, Belgium; 9 Present address: Research Foundation – Flanders (FWO), Egmontstraat 5, 1000, Brussels, Belgium

**Keywords:** Social capital, Health inequity, Health gradient, Neighbourhoods, Children, Adolescents

## Abstract

**Background:**

Although most countries in the European Union are richer and healthier than ever, health inequalities remain an important public health challenge. Health-related problems and premature death have disproportionately been reported in disadvantaged neighbourhoods. Neighbourhood social capital is believed to influence the association between neighbourhood deprivation and health in children and adolescents, making it a potentially interesting concept for policymakers.

**Methods:**

This study aims to review the role of social capital in health inequalities and the social gradient in health and well-being of children and adolescents. A systematic review of published quantitative literature was conducted, focussing on (1) the mediating role of neighbourhood social capital in the relationship between socio-economic status (SES) and health-related outcomes in children and adolescents and (2) the interaction between neighbourhood social capital and socio-economic characteristics in relation to health-related outcomes in children and adolescents. Three electronic databases were searched. Studies executed between 1 January 1990 and 1 September 2011 in Western countries (USA, New Zealand, Australia and Europe) that included a health-related outcome in children or adolescents and a variable that measured neighbourhood social capital were included.

**Results:**

Eight studies met the inclusion criteria for the review. The findings are mixed. Only two of five studies confirmed that neighbourhood social capital mediates the association between neighbourhood deprivation and health and well-being in adolescents. Furthermore, two studies found a significant interaction between neighbourhood socio-economic factors and neighbourhood social capital, which indicates that neighbourhood social capital is especially beneficial for children who reside in deprived neighbourhoods. However, two other studies did not find a significant interaction between SES and neighbourhood social capital. Due to the broad range of studied health-related outcomes, the different operationalisations of neighbourhood social capital and the conceptual overlap between measures of SES and social capital in some studies, the factors that explain these differences in findings remain unclear.

**Conclusions:**

Although the findings of this study should be interpreted with caution, the results suggest that neighbourhood social capital might play a role in the health gradient among children and adolescents. However, only two of the included studies were conducted in Europe. Furthermore, some studies focussed on specific populations and minority groups. To formulate relevant European policy recommendations, further European-focussed research on this issue is needed.

## Background

### Health inequality and the social gradient in health

Health is distributed unevenly within the European population. Ill health and premature death increase with a declining social position [1,2]. Such variation is evident between richer and poorer countries, within countries [3,4] and within the geographic unit of a city. For instance, the life expectancy of a baby born in the most affluent neighbourhoods of Glasgow (Scotland) is approximately 10 years longer than that of its counterpart born in the most disadvantaged parts of the city [5]. In all age groups, people from lower socio-economic strata suffer a heightened health risk for nearly all diseases [6]. However, the detrimental effect of socio-economic characteristics on health extends beyond the existence of a health gap between the lowest and highest socio-economic groups. The relationship between socio-economic status and health status typically follows a monotonic course: health differences are gradually found between all rungs of the socio-economic ladder. People who are at a specific position on this proverbial ‘ladder’ systematically tend to have worse health than those one rung above them and tend to have better health than those one rung below them. This phenomenon is referred to as the social gradient in health [7,8] and implies that socio-economic factors influence the health of the entire population.

Socio-economic factors are related not only to health, but also to social resources, such as social networks, social support and trust [9,10]. As Michael Marmot states, “the pattern of social relationships follows a social gradient” [11]. People who are on the lower rungs on the proverbial social ladder generally have less diverse social networks and more often report a lack of social support [11,12]. Furthermore, community SES is positively related to levels of trust and social capital in communities [4].

The next section will first focus on the literature on health inequalities and the social gradient in children and adolescents and the difficulties underlying this theme. Next, the influence of socio-economic characteristics on the health of children and adolescents will be explored, including the respective role of family and neighbourhood socio-economic factors. Subsequently, this introduction will explore the role of neighbourhood social capital in health inequalities among children and adolescents.

### Health inequality and the social gradient in the health of children and adolescents

Numerous studies [1,13,14] describe the existence of health inequalities and the social gradient in the health of adults. These findings are, to some extent, also confirmed for children and adolescents [15-19]. Various studies describe the presence of a social gradient for child mortality [13,20] and morbidity [13,21], self-reported health [3,22], health complaints [23], eating habits [24], healthy eating, sedentary behaviour and overweight [13,25] and bullying exposure [26].

However, the evidence on health inequalities among children and adolescents is inconsistent [23,27,28]. This is especially the case for health inequalities among older children and adolescents [28,29], as the influence of socio-economic factors on the health of children and adolescents is thought to vary with age [21]. Some authors state that the social gradient diminishes as children age, thereby disappearing in adolescence [23,29]. Other authors present opposite findings, namely, a rise in the negative association between socio-economic factors and health in adolescence [30,31]. Exploring the association between SES and health in adolescents is particularly challenging due to the complexity of assigning SES to people in this age group. Reasons for this complexity include, among others, methodological problems (e.g. adolescents are not always able to adequately report parental income, occupation or education) [28], and the rising importance of adolescents’ own educational level and social network (e.g. effects of peer groups in general and professional education) [29]. The differential effect of SES on health throughout the course of life might be explained by the dynamic impact of the mediating factors on the relationship between socio-economic factors and health outcomes throughout childhood and adolescence. For instance, emotional and cognitive mediators, such as depressive feelings, and neighbourhood and social factors, such as peer influence and youth culture, play a more pronounced role in late childhood and adolescence [21,23,32].

### Family- and neighbourhood-level socio-economic factors and their impact on the health and well-being of children and adolescents

The association between socio-economic characteristics and health status is a consistent finding in the social sciences [33-37]. Neighbourhood socio-economic characteristics contribute to the explanation of social inequalities in health [38]. Furthermore, the socio-economic conditions that individuals experience during childhood have a considerable influence on their later life; inequalities in childhood have both direct and long-term negative effects [39-41].

It is generally assumed that the relationship between neighbourhood characteristics and outcomes in children and adolescents is mainly indirect [42,43]. Researchers have suggested various pathways in an attempt to explain the relationship between neighbourhood SES and health, resulting in three theoretical models. Researchers who follow a neo-material vision believe that the association between neighbourhood SES and health can be attributed to a differential access to material resources [10,44]. *The institutional resources model* by Leventhal and Brooks-Gunn [42] claims that the quality, accessibility and availability of institutional resources might explain the relationship between neighbourhood characteristics and outcomes in children and adolescents. Following this model, the quantity and quality of resources that affect the lives of young people (e.g. child care, leisure time activities, education, health care facilities) are likely to be lower in neighbourhoods with high levels of disadvantage (low SES, high ethnic diversity, high residential instability). For instance, research in various cities in the USA found that the quality of the sidewalks was lower in high-poverty neighbourhoods than in low-poverty neighbourhoods, which is in turn believed to negatively influence children’s physical activity [45].

As an alternative pathway, researchers have emphasised the importance of psychosocial pathways that link socio-economic deprivation to worse health [10,44,46]. According to *the relationships model*[42,47], the home environment, parental networks and parental characteristics mediate the influence of neighbourhood characteristics on youth’s outcomes. More specifically, levels of parental characteristics that enhance child well-being (e.g. parental social support, parental monitoring and other qualitative parenting practices) are lower in deprived neighbourhoods compared to non-deprived neighbourhoods, whereas levels of harmful parental characteristics (e.g. parental stress, exposure to intra-family violence) are higher in deprived neighbourhoods [47]. Furthermore, *the norms and collective efficacy model* states that neighbourhood structural disadvantage negatively influences the neighbourhood’s social norms. Disadvantaged neighbourhoods are believed to have fewer health-promoting social norms and a lower willingness to intervene for the common good, which in turn has a negative effect on child and adolescent outcomes [42,47]. This finding is in line with research that ascribes a part of the association between macro-level income inequality and health to a decline in the collective social fabric [4,8,48-50].

The socio-economic conditions in the neighbourhood and family are important for children and adolescents’ health and well-being. Access to goods and resources, on the one hand, and parental psychopathology or parenting practices, on the other hand, are believed to explain the link between family SES and the health of children and adolescents [51]. An interplay between family- and neighbourhood-level socio-economic factors is also evident. On the one hand, better socio-economic circumstances in neighbourhoods are related to, among other factors, higher quantity and quality of neighbourhood institutional resources and more supportive family and neighbourhood social processes, which results in better child and adolescent outcomes net of the influence of socio-economic factors at the family level. On the other hand family socio-economic circumstances are believed to affect the influence of community SES on the health of children and adolescents via a multiplicative effect. In other words, residing in a context in which one’s family socio-economic background is relatively advantaged or deprived compared with the general socio-economic background of the neighbourhood might contribute to negative health outcomes. For instance, Gordon et al. [51] find that ADHD is more frequently present when the socio-economic background of the family and the neighbourhood are dissimilar.

The current study explores the role of neighbourhood social capital in the health gradient among children and adolescents. First, the concept of ‘social capital’ is examined, with attention to how this diffuse concept is defined in the current study. Second, the relationship between social capital and the health of children and adolescents is investigated.

### Social capital: the concept explored

Social capital refers to the idea that social networks are a potential resource for individuals, communities and society as a whole [52]. Pierre Bourdieu, James Coleman and Robert Putnam are considered to be the founding fathers of the conceptualisation of social capital [52,53]. They interpret this concept from diverse perspectives, as they study the concept from varying theoretical backgrounds [54]. Bourdieu’s conceptualisation of social capital can be fit into an overarching theory on social stratification. He defines the concept as “the aggregate of the actual or potential resources which are linked to possession of a durable network of more or less institutionalised relationships of mutual acquaintance or recognition” [55]. With this definition, he identifies social networks and the resources within social networks as being the core elements of social capital [53,56]. Bourdieu’s relational definition of social capital is in contrast to the normative approach to social capital of Putnam and Coleman [53,56]. Putnam refers to social capital as “features of social organisation such as networks, norms, and social trust that facilitate coordination and cooperation for mutual benefit” [57]. This definition of social capital is the most widely cited in health research, but it has been criticised [56,58].

The lack of conceptual clarity has persisted in the social capital literature; social capital is used to refer to a vast array of social characteristics [59]. Therefore, discussions and disagreements are present at many levels in social capital research [60]. A common criticism is that social capital has been used to address so many social determinants that the term has lost all heuristic value [60,61]. This lack of consistency regarding the use of social capital is reflected in the lack of clarity on how to measure the concept and in the variety of constructs and labels that are used to refer to neighbourhood social capital (e.g. social support, social resources, social cohesion, informal social control) [62]. However, one of the most important discussion points in the literature is the level at which social capital has an influence in general [63] and, more specifically, on health outcomes [52,64]. Whether social capital is a societal construct rather than a characteristic of individuals is still a subject of debate [60,64].

There is a growing research interest in how places affect people’s health and well-being. Researchers previously focussed on structural and sociodemographic characteristics of local areas. During the last decennia however, attention shifted to include social processes as well [37]. The present review focusses on social capital at the level of local communities. 

The exact meaning of a community is an ongoing debate [65,66]. 

Researchers have used the term to refer to “a collection of individuals characterised by dense, cross-cutting networks” [67]. The term ‘community’ includes the individual and subjective meaning that people assign to the place in which they live, work and learn, which makes it challenging to operationalise the concept [68]. Researchers often turn to more delineated, geographically distinct methods of operationalising communities, such as census tracts and neighbourhood blocks, among others, to facilitate data collection.

### Social capital and health

The present research conceptualises social capital as a collective characteristic of places that arises from people’s shared experiences [69]. This study focusses on the potential role of neighbourhood social capital in the social gradient found in children and adolescents’ health and well-being. Increasing evidence indicates that social capital has a positive influence on various aspects of people’s physical and mental health [70-73]. Most studies on the influence of social capital on health have focussed on adult populations [74-76]. However, several studies have identified the protective effect of social capital on diverse health outcomes in children and adolescents, such as self-rated health [77-80], physical and psychological health complaints [81,82] and health behaviour [83-85].

Although many authors have reported the beneficial influence of social capital, the possible negative effects of social capital are also identified [53,86-88]. Portes (1998) described four negative consequences of social capital. First, strong social bonds within a group might prevent others from joining the network, thereby leading to the exclusion of ‘outsiders’. Second, social capital – and the resulting levels of social norms and social control – might be demanding and place large claims on group members. Third, high social capital might restrict the individual freedom of the members due to the rising demands for conformity to the group. Finally, social capital can foster downward levelling norms that ‘trap’ individuals within the group.

### Objectives

This literature review is conducted as a part of the European research project *The Gradient*, coordinated by Eurohealthnet. This project (April 2009 – 2012) aims to address the knowledge gap concerning which actions are effective to level the social gradient in health among children and adolescents in Europe. Health inequalities are currently regarded as a public health challenge of utmost importance in the EU. This project aspires to produce guidelines that will influence policymakers in their efforts to tackle these inequalities [41,89].

Childhood experiences are known to contribute to health inequalities in adulthood [1,81,90,91]. The explanation of health inequalities has undergone a vast evolution over the past several years. Initially, an explanation was sought in the higher prevalence of health-risk behaviour in people lower on the social ladder. Later, the focus shifted to the material deprivation of people with a low SES, with obvious consequences for housing, access to services, employment, etc. However, both a focus on individual behaviour and a focus on material deprivation fail to grasp the complex reality of health inequity. Recently, a shift towards economic and social macro factors has gained growing attention. For instance, research has shown that income inequality is related to health both within and between countries [4,92]. This association is hypothesised to operate via a deterioration of social capital [71,93-97]; however, the evidence remains limited and mixed [98]. A large part of the evidence that explores health inequalities focusses on adults [93] and/or investigates the inequity between extensive geographic areas (i.e., countries or states). This review can be positioned in the literature that turns to local social processes to explain the link between socio-economic characteristics and health [47].

**Figure 1:**
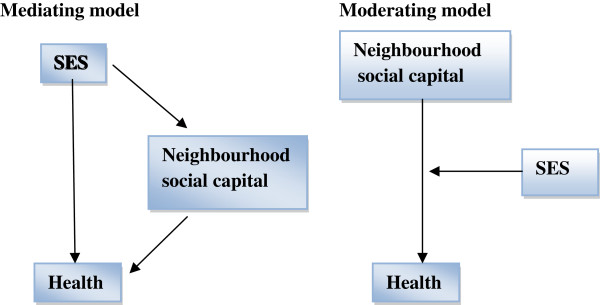
**Expected mediation and moderation model.** SES: socio-economic status.

It aims to explore the role of neighbourhood social capital in the relationship between both individual and neighbourhood-level socio-economic factors and health in children and adolescents. More specifically, the first research aim is to investigate whether components of neighbourhood social capital have a mediating or moderating effect on the relationship between SES and health-related outcomes in children and adolescents (see Figure 1).

In a mediation model, a mediating variable is hypothesised to be intermediate in the relation between an independent variable and an outcome measure. This study focusses on the mediating effect of neighbourhood social capital on the association between socio-economic factors and health in children and adolescents, which is in line with the *relationships model* and the *norms and collective efficacy model* by Leventhal & Brooks-Gunn [42,47].

Social capital theory does not include hypotheses on potential moderating effects involving social capital. However, research on adults has indicated the interplay between socio-economic factors and social capital. On the one hand, it is possible that access to social capital is particularly helpful for people with fewer socio-economic resources, as it compensates for their low personal capital *(compensation effect proposition).* On the other hand, personal and social capital might reinforce each others’ influence on health, leading to a greater impact of social capital for people with a high SES *(cumulative advantage proposition)*[56,99]. The second aim of this literature review is to analyse the interplay between socio-economic factors and neighbourhood social capital in relation to the health and well-being of children and adolescents. To test whether the association between an independent variable and an outcome measure differs across levels of a third variable, a moderation model must be analysed. A moderator variable affects the strength and/or direction of the correlation between a predictor and an outcome, i.e., enhancing, reducing, or changing the influence of the predictor.

## Method

For the purpose of inclusion, the literature review identified all observational and intervention studies (published between 1 January 1990 and 1 September 2011) that considered neighbourhood social capital to be a mediating or a moderating factor in the relationship between socio-economic status and the health of children or adolescents. The following criteria for inclusion were established: studies had to include a health-related outcome, a variable proposed to measure neighbourhood social capital, a measure of socio-economic conditions and had to focus on children and/or adolescents. The selection of search terms that were considered to be components of social capital was based on published theoretical literature on social capital in health research [75,76,100-102]. An overview can be found in Table 1. To be included, these variables had to either be measured directly at the neighbourhood level or the individual scores for these variables had to be aggregated to a neighbourhood score. Socio-economic status was measured on the basis of income, education, employment, belongings, or family structure and could either be measured at the individual/family level or at the neighbourhood level.

**Table 1:** Overview of search terms

**N°**	**Collective terms**	**Search terms**
1	*Components of Social Capital*	social capital OR social support OR social resources OR social cohesion OR neighborhood cohesion OR neighbourhood cohesion OR informal social control OR collective efficacy OR neighborhood disorder OR neighbourhood disorder OR social disorganisation OR social disorganization OR social networks
2	*Components of Health Gradient*	gradient OR socioeconomic factors OR inequity OR health disparities
3	*Components of SES*	socioeconomic status OR social class OR neighborhood socioeconomic disadvantage
4	*Components of neighbourhood*	residence characteristics OR neighborhood OR neighbourhood
5	*Population of young people*	infant OR child OR adolescent OR newborn infant OR preschool child
6	*Full search string*	#1 AND (#2 OR #3) AND #4 AND #5

Table 1 Overview of search terms at the basis of the search strategy.

The review focusses on health-related outcomes in children or adolescents. The outcome measures of interest were not specified in the search strategy. The relevance of the studies’ outcome measures was assessed during the selection process. Studies on academic achievement, language deficiency and domestic violence were excluded. “Academic achievement” and “education” were exclusion criteria because they are not indicators of health or well-being; rather, they are important determinants of health and indicators of socio-economic status. Studies on the cognitive development of small children (unrelated to an academic/school context) were included, as cognitive development is closely related to children’s well-being [103]. “Language deficiency” is considered to be an exclusion term when referring to speech problems, that is, disabilities that affect people’s language skills (e.g. motor disabilities or stuttering).

Regarding methodological and statistical approaches, the review focussed on quantitative studies that used statistical analyses appropriate to investigate the mediating and/or moderating effect of social capital on the relationship between SES and health-related outcomes. To test for mediation, studies were expected to (1) use path analyses (such as structural equation modelling), (2) use a direct test of the indirect effect of SES on health of children and adolescents via neighbourhood social capital (e.g. the product of coefficients test or Sobel test [104]) or (3) enable the analysis of the influence of the introduction of neighbourhood social capital on the relationship between SES and health. This last method is related to the most widely used method to detect mediation, the causal steps approach introduced by Baron & Kenny [105]. By estimating different pathways between the dependent, independent and mediator variable, this approach attempts to indirectly test mediation. However, recently, this method has strongly been criticised [106,107]. Therefore, we did not expect studies to strictly follow the causal steps approach by Baron & Kenny [105].

To test for moderation, studies were expected to analyse the interaction between SES and neighbourhood social capital.

The included studies were further restricted to those that include a general non-clinical population of children and adolescents of 0 to 18 years of age. The age groups included in this review range from newborn to adolescent (0–18 years). We employed the term “children” for the age group 0–12 years and “adolescents” for the age group 12–18 years, in accordance with the Glossary composed for the Gradient Project. In cases in which the age group of a study crossed this delineation, we used the term that refers to the oldest children in the study.

The delineation of a sample to specific geographic or socio-demographic areas (e.g. deprived neighbourhoods, rural areas) or specific age, gender, socio-economic or racial groups was not considered to be a reason for exclusion. The included studies were further delimited to studies executed in Western countries (USA, New Zealand, Australia and Europe), published in peer-reviewed journals and written in English, French, Dutch, German, Spanish, Icelandic or Czech.

### Search strategy

Three large and comprehensive electronic databases - PubMed, Web of Knowledge and Sociological Abstracts - were searched for relevant publications, using the search strategy presented in Table 1. Of note, this is a simplified version of the search strategy, as each database has a customised search string dependent on the number of hits of each unique search term, the combination of search terms and the thesaurus.

### Procedure and flowchart

**Figure 2:**
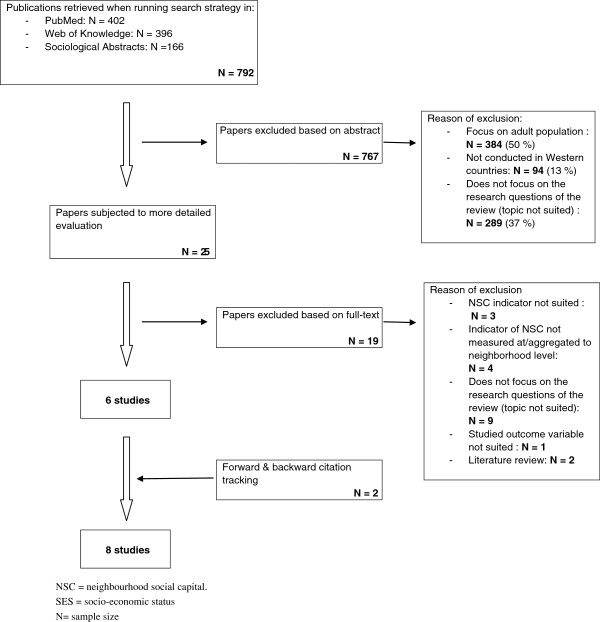
**Flowchart of the selection process.** NSC = neighbourhood social capital. SES = socio-economic status. N= sample size.

A flowchart of the selection procedure is presented in Figure 2. The search strategy resulted in the identification of 792 articles. These articles were all screened by a first reviewer (BDC).

First, the abstracts of all of the retrieved articles were screened using the inclusion and exclusion criteria. Articles that met one of the exclusion criteria (*N* = 767) were excluded and sorted by reason of exclusion (i.e., based on population, region or topic). Half of the papers were excluded because the research populations were not children or adolescents. Additionally, 13% were excluded because the region was beyond the scope of this review and another 37% were off-topic. A second reviewer (VV) performed a random search of one-fourth of the 792 originally identified papers. No differences in the selection of studies were found between the 25% sample and the entire search. The articles that did not explicitly meet one of the exclusion criteria (*N* = 25) were selected for a detailed evaluation during the first selection round. Their relevance was evaluated by two independent researchers (BDC and LM). These selections were then compared and discussed until a consensus was reached. The researchers disagreed on a minimal number of articles. The most important reason for exclusion during this phase of the selection process was that studies did not investigate the interplay between socio-economic status and neighbourhood-level social capital on health-related outcomes in children and adolescents.

Based on full-text articles, a final selection was made (*N* = 6). This selection was mainly based on the appropriateness of the methods used to answer the current research questions.

All of the retrieved articles were independently screened by a second reviewer (VV). Both selections were compared and discussed until a consensus was reached. Citation tracking was used to identify additional studies from the reference lists of previous (backward citation tracking) and future (forward citation tracking) relevant studies. Potentially eligible studies were identified by a reviewer (BDC) who scanned titles and abstracts. When there was uncertainty about potential relevance, a second reviewer (VV) read the abstracts, allowing a joint decision to be made. Full-text papers of all potentially eligible studies were obtained to enable data extraction. The first reviewer (BDC) selected two relevant studies, which were also reviewed and approved by the other reviewers (VV and LM). Finally, eight studies were included.

### Description and quality of included studies

For each included study, a data-extraction form and a quality assessment form were completed. The data-extraction form was developed based on a review of social capital and well-being in children [102] and a review of obesity and food insecurity by the Effective Public Health Practice Project [108]. The quality assessment tool was based on a tool developed by the Effective Public Health Practice Project and used by Mirza et al. [108]. The original tool (downloadable at http://www.ephpp.ca/tools.html) was adapted because it was developed to evaluate intervention studies. The original tool contained, among others, evaluation criteria on the processes of blinding and on intervention integrity, which were not useful in evaluating observational studies. Adaptations to the original tools were made in collaboration with the research partners to match the specific character of the studies of interest. Both forms were completed by two independent researchers. Later, the forms were compared and decisions were discussed until a consensus was reached. Subsequently, the scores were evaluated by a sociologist with experience in multilevel modelling. The following six domains concerning research quality were evaluated: selection bias, allocation bias, confounders, data collection methods, withdrawals and dropouts and analysis. For all domains, except for ‘analysis’, a summary score, ranging from weak, to moderate and strong, could be calculated. The analysis of the study was evaluated based on structured questions from the developed tool and extra comments by the methodological expert. The quality assessment tools were only based on the content of the published papers, without contacting the authors for further information.

### Analysis of study findings

A narrative review was conducted. A meta-analysis was not attempted due to the heterogeneity of populations and outcome measures among the included studies. Findings were compared according to the investigated effect (moderating or mediating).

## Results

A search of the published literature identified a total of eight research articles that met the inclusion criteria. None of the articles were intervention studies. Only two studies were conducted in Europe, four were conducted in the USA and two were conducted in Canada. Six studies had a multilevel design. A summary of the included studies and the results is presented in Table 2.

**Table 2:** Description of the included studies

**Reference**	**Region**	**Population**	**SES**	**Social capital**	**Outcome**	**Mediating/pathway model**	**Moderating model**
Kohen, Brooks-Gun, Leventhal, & Hertzman, 2002	Canada	Children (4–5 y)	Neighbourhood income, neighbourhood family structure, neighbourhood unemployment rate	Neighbourhood cohesion *(N items = 5, IR: α=0.87)*	Children’s receptive verbal ability + behaviour problems	*Model tested in the study, but no significant results found*	*Model not tested in the study*
Xue, Leventhal & Brooks-Gunn et al., 2005	USA, Chicago	Children (6–12 y)	Neighbourhood concentrated disadvantage, family income, maternal education and employment	Neighbourhood collective efficacy: informal social control *(N items = 5, IR: not reported)* + social cohesion *(N items = 5, IR: not reported)*, neighbourhood organisational participation *(N items = 7, IR: not reported)*	Mental health problems (internalising problems)	Neighbourhood concentrated disadvantage → neighbourhood collective efficacy → mental health problems	*Model not tested in the study*
Caughy & O’Campo, 2006	USA, Baltimore	African American children (3 – 4.5 y)	Economic impoverishment: poverty rate, unemployment, vacant housing, single-headed families	Parental psychological sense of community *(N items = 10, IR: α=0.92)*, parental willingness to assist children in need (*N items = not reported, IR: α=0.81)* and stop acts of misbehaviour *(N items = not reported, IR: α=0.85)*	Child cognitive competence	*Model tested in the study, but no significant results found*	*Model not tested in the study*
Drukker, Kaplan, Schneiders, Feron, & van Os, 2006	The Netherlands, Maastricht	Adolescents (Age M wave 1=10.2 y, wave 2 = 13.5 y)	Neighbourhood social disadvantage index (contains information on family structure, employment status, social benefits, ethnicity, voting behaviour and income).	Collective efficacy: informal social control, social cohesion and trust *(N items and IR not reported)*	Quality of life: self-esteem and satisfaction	*Model not tested in the study*	*Model tested in the study, but no significant results found*
Kohen, Leventhal, Dahinten, & McIntosh, 2008	Canada	Children (4–5 y)	Neighbourhood structural disadvantage: income, education, unemployment, family structure	Neighbourhood cohesion *(N items=5, IR not reported)*	Verbal ability + behaviour problems	SES -> neighbourhood cohesion -> maternal depression -> punitive parenting -> behaviour problems	*Model not tested in the study*
						SES -> neighbourhood cohesion -> family functioning -> consistent parenting -> verbal ability	
Caughy, Nettles & O'Campo, 2008	USA, Baltimore	Children 6–7 y	Neighbourhood concentrated economic disadvantage, parental educational attainment, parental employment status	Neighbourhood potential for community involvement with children *(N items=not reported, IR: α=0.78 (individual level) and 0.95 (neighbourhood. level)*, neighbourhood negative social climate (*N items=not reported, IR: α=0.76)*	Child behaviour problems (internalising and externalising behaviour problems)	*Model not tested in the study*	Neighbourhood concentrated economic disadvantage X neighbourhood potential for community involvement with children
Karriker-Jaffe, Foshee, Ennett, & Suchindran, 2009	USA	Rural adolescents (11–18 y)	Neighbourhood socio-economic disadvantage score: education, employment, economic resources	Neighbourhood-level social organisation: neighbourhood social bonding (*N items=4, IR: α=0.75)*, neighbourhood social control (*N items=6, IR: α=0.91)*	Aggression trajectories	*Model tested in the study, but no significant results found*	*Model tested in the study, but no significant results found*
Odgers et al., 2009	England & Wales	Children 5–10 y	Neighbourhood deprivation versus affluence, family socio-economic disadvantage	Neighbourhood collective efficacy *(IR neighbourhood level: α=0.88):* consists of informal social control (*N items=5)* + social cohesion (*N items=5*)	Children’s antisocial behaviour: aggression + delinquency	*Model not tested in the study*	Neighbourhood deprivation versus affluence X neighbourhood collective efficacy
*Total number of studies*	*8*						

y = years of age; M= Mean; IR= Internal reliability; N items = Number of items in scale.

### Quality of included studies

For each included study, a quality assessment tool was completed to evaluate the overall quality of the study design and analysis. The results of the quality assessment are presented in Table 3. Overall, the quality of the studies was mostly moderate to strong.

**Table 3:** Quality assessment of the included studies

	**Selection bias**	**Allocation bias**	**Confounders**	**Data collection methods**	**Withdrawals and dropouts**	**Comment on the analysis**
Kohen, Brooks-Gun, Leventhal, & Hertzman, 2002	S	M	S	S	NA	-No power calculation
						-Results unambiguously reported
						-Handling of missing data not reported
						-Inappropriate statistical methods: multilevel model is required to answer research question
						-Risk of clustering of children within the same families
Xue, Leventhal & Brooks-Gunn et al., 2005	M	M	S	S	NA	-ICC calculated
						-No power calculation
						-Results are unambiguously reported
						-Appropriate handling of missing data
						-Appropriate statistical methods with remarks:
						-Analysis of level 1 and 2 variances reported
Caughy & O’Campo, 2006	M	M	M	S	NA	-ICC calculated
						-No power calculation
						-Results unambiguously reported
						-Appropriate statistical methods with remarks
						-No level 1 predicators entered in the model
						-Analysis of level 2 variance not reported
						-Small N
						-Handling of missing data not reported
Drukker, Kaplan, Schneiders, Feron, & van Os, 2006	M	M	S	S	M	-ICC calculated
						-No power calculation
						-Results are partially unambiguously reported
						-Appropriate statistical methods with remarks
						-Possible selective drop-out
						-Analysis of level 1 and level 2 variance not reported
						-Small N
						-Handling of missing data not reported
Kohen, Leventhal, Dahinten, & McIntosh, 2008	S	M	S	S	NA	-No power calculation
						-Results are unambiguously reported
						-Appropriate statistical methods with remarks
						-Multilevel SEM would be more suited
						-Not possible to assess level 1 and level 2 variance, calculate changes in r^2^, etc.
						-Appropriate handling of missing data
Caughy, Nettles & O'Campo, 2008	W	M	S	W	NA	-ICC not calculated
						-No power calculation
						-Results are unambiguously reported Appropriate statistical methods with remarks:
						-Small N
						-No analysis of level 1 and 2 variances reported
Karriker-Jaffe, Foshee, Ennett, & Suchindran, 2009	M	M	S	S	W	-ICC calculated
						-No power calculation
						-Results are unambiguously reported
						-Appropriate statistical methods with remarks
						-No analysis of level 1/level 2 variance
						-Appropriate handling of missing data
Odgers et al., 2009	M	M	S	S	NA	-No power calculation
						-Results are unambiguously reported
						-Appropriate handling of missing data
						-Appropriate statistical methods

W=weak; M=moderate; S=strong.

NA = not applicable; ICC = intraclass correlation coefficient; N = sample size; SEM = Structural Equation Modelling.

### Measures of socio-economic characteristics

The indicators used to measure socio-economic characteristics at the neighbourhood, family or individual level varied across studies. Measures on income, poverty or employment status were used in all of the included studies to operationalise socio-economic status. Furthermore, socio-economic status was most frequently measured using variables on parental educational attainment [109-112] and family-structure [113-116]. All of the studies included measures of neighbourhood socio-economic factors, and three of the included studies measured socio-economic characteristics at the family level [110,112,116].

### Measures of social capital

The indicators used to measure social capital were also diverse. The most common ways to operationalise the complex concept of ‘social capital’ were through forms of social control or collective efficacy. All studies included a measure that refers to inhabitants’ willingness to intervene in case of neighbourhood problems and the extent to which the inhabitants would jointly attempt to find solutions to neighbourhood problems. However, the terms used to refer to this idea included ‘informal social control’ [110,115,116], ‘collective efficacy’ [110,111,113,115,116], ‘social cohesion’ [111,113], ‘neighbourhood potential for community involvement with children’ [112] and ‘willingness to stop acts of misbehaviour’ [114]. Six of the included studies [109-111,113,115,116] used (parts of) the same scale, developed by Sampson and colleagues [117]. Furthermore, diverse indicators of social capital were used, including social bonding [109] and organisational membership [110].

All studies utilised a scale or multiple variables to measure social capital.

### Outcome measures

All studies included measures of well-being as outcome variables: behaviour problems [109,111-113], verbal ability [111,113], mental health problems [110], self-esteem and satisfaction [115] and cognitive abilities [114].

### Types of neighbourhoods

Most of the neighbourhoods were assumed to be representative of national neighbourhoods in terms of socio-economic factors, as they were randomly selected from city-wide or state-wide populations or included entire populations. However, Karriker-Jaffe et al. [109] notice that their neighbourhoods systematically had a lower median household income and lower median housing value than the general USA population. Furthermore, in some studies, the proportion of African American residents in the included neighbourhoods was high [112,114]. None of the studies solely focussed on deprived neighbourhoods.

### Used analyses

Five studies investigated the mediating role of social capital in the association between socio-economic factors and children and adolescent’s health-related outcomes [109-111,113,114]. One of these studies explored the pathways by which socio-economic factors influence children’s outcomes via social capital [111]. Four included studies tested for a moderating effect of social capital [109,112,115,116].

### Results on the mediating effect of social capital in the relationship between SES and health

Most of the studies that analysed the role of social capital as a mediator in the association between socio-economic factors and health focussed on children. Caughy & O’Campo explored the mediating effect of the inhabitants’ willingness to assist children in need on the association between neighbourhood impoverishment and children’s cognitive competence [114]. The analyses did not support the hypothesis of neighbourhood social organisation as a mediating variable. Kohen and colleagues [118] focussed on children’s verbal abilities and behaviour problems in two papers using longitudinal data. First, they investigated the mediating effect of components of social capital on the relationship between neighbourhood socio-economic factors and children’s verbal ability and behaviour problems. A mediating effect of social capital on the relationship between neighbourhood socio-economic factors and behaviour problems was not found. In their second study [111], the authors explored how neighbourhood processes affect young children using structural equation modelling. Two significant pathways between neighbourhood disadvantage and children’s outcome measures that include neighbourhood social cohesion were found. Neighbourhood disadvantage had a significant indirect negative effect on children’s behaviour problems via its influence on less neighbourhood cohesion, higher maternal depression and more punitive parenting. The second significant indirect negative effect of neighbourhood disadvantage on children’s verbal ability was through less neighbourhood cohesion, worse family functioning and less consistent parenting. In this study, neighbourhood social cohesion referred to the social organisation in the neighbourhood, with a focus on social control and collective efficacy in the neighbourhood. Xue and colleagues [110] investigated the effect of neighbourhood social processes on mental health problems. The data of this cross-sectional study support the authors’ hypothesis that neighbourhood collective efficacy mediates the relationship between neighbourhood economic disadvantage and higher internalising problems in children aged 5–11 years.

The study by Karriker-Jaffe and colleagues [109] is the only included study that explored social capital’s mediating role in the relationship between socio-economic factors and adolescents’ health. In a multilevel longitudinal study, they focussed on the aggression trajectories of rural adolescents between 11 and 18 years of age. Neighbourhood social bonding and neighbourhood social control were hypothesised as mediating variables in the association between neighbourhood disadvantage and aggression. However, the findings did not support this hypothesis.

### Results on the moderating effect of social capital in the relationship between SES and health

Two studies focussed on children in analysing social capital’s moderating role in the association between socio-economic factors and health. Caughy and colleagues [112] examined the role of neighbourhood social processes at differing levels of neighbourhood deprivation. They found that high levels of neighbourhood potential for community involvement with children (referring to levels of collective efficacy and social cohesion) were associated with less behaviour problems in children in economically deprived neighbourhoods. A similar association was not found for children in neighbourhoods with a higher socio-economic status. Odgers and colleagues focussed on the association between neighbourhood collective efficacy and children’s antisocial behaviour [116]. The data suggested that higher levels of neighbourhood collective efficacy were negatively associated with the rate of aggressive and delinquent behaviour at school entry only for children living in deprived neighbourhoods. Two other studies focussed on older children and adolescents’ health in this context. The study by Drukker and colleagues [115], which was one of the two included European studies, had a longitudinal design. It explored neighbourhood social cohesion and trust as a moderating variable in the influence of neighbourhood social disadvantage on changes in self-esteem in adolescents between a baseline (mean age = 11.2) and follow-up (mean age = 13.5) measurement. Neighbourhood social disadvantage (measured by an index including income, ethnicity, family structure and occupational status) was associated with a statistically significant positive evolution of self-esteem in adolescents with lower-educated parents, but with a negative evolution of self-esteem in adolescents with higher-educated parents. Both the positive association in adolescents of lower-educated parents and the negative association in adolescents of higher-educated parents between social capital and self-esteem were stronger in neighbourhoods with low levels of social cohesion and trust. This evidence is, however, statistically weak given that the interaction between neighbourhood disadvantage and social capital did not reach statistical significance (p = 0.13). In their multilevel longitudinal study, Karriker-Jaffe and colleagues [109] did not find support for their hypothesis that neighbourhood social bonding and neighbourhood social control serve as moderating variables in the association between neighbourhood disadvantage and aggression in rural adolescents between 11 and 18 years of age.

## Discussion

### Summary of results

To analyse the role of neighbourhood social capital in the relationship between socio-economic status and health-related outcomes in children and adolescents, a review of the published literature was conducted. First, we examined neighbourhood social capital as a mediator in the association between socio-economic status and health in children and adolescents and the pathways that underlie this association. Two of the included studies found that social processes in the neighbourhood (referred to as ‘social cohesion’ and ‘collective efficacy’) mediate the association between neighbourhood disadvantage and health-related outcomes in children (aged between 4 and 12) [110,111]. Furthermore, Canadian research found that the relationship between neighbourhood deprivation on the one hand and verbal ability and behaviour problems in young children on the other hand runs through social processes at home, such as maternal mental health and parenting practices [111]. However, three other studies did not find significant results when analysing neighbourhood social capital as an intermediate variable in the relationship between socio-economic factors and health in young children (aged 3 to 5) [113,114] and adolescents [109]. As such, this review finds partial support for the *norms and collective efficacy model* and full support for the *relationships model* put forward by Leventhal & Brooks-Gunn to explain how neighbourhoods influence outcomes in children and adolescents [42,47]. Furthermore, the current study investigated the interaction between neighbourhood social capital and socio-economic characteristics in explaining the health of children and adolescents. Two studies found that the relationship between neighbourhood social capital and behaviour problems in children (aged 5–10 years) depends on the socio-economic characteristics of the neighbourhood. In both studies, neighbourhood social capital was only associated with lower levels of problematic behaviour for children in deprived neighbourhoods [112,116]. Two other studies, which focussed on aggression and quality of life in adolescents, did not find a significant interaction between neighbourhood social capital and SES [109,115]. This finding partially supports the “compensation effect proposition”, which states that social capital is particularly beneficial for people with low levels of personal capital (e.g. low income, low educational level) [56,99].

The eight studies that met the inclusion criteria utilised diverse health-related outcomes and social capital indicators. Due to the diverse indicators used to measure both social capital and health, it is challenging to draw firm conclusions from this study. However, the results suggest that components of neighbourhood social capital such as neighbourhood social cohesion and neighbourhood social control influence the impact of socio-economic factors on health outcomes in children. Furthermore, it seems likely that neighbourhood social capital is especially beneficial for children who reside in deprived neighbourhoods, although additional research is needed to support this notion.

Most of the included studies focussed on young children and their parents. Of the six studies that focussed on pre-school and school-aged children, four identified neighbourhood social capital as a significant mediator in the relationship between socio-economic status and health in children or found that neighbourhood socio-economic status moderates the association between neighbourhood social capital and health in children. In contrast, neither of the two studies that focussed on adolescents found a significant interaction between socio-economic factors and neighbourhood social capital. This result could indicate that neighbourhood social capital is more important for the health of younger children, which contradicts earlier findings by Chen et al. [119]. Although further research is needed to confirm this finding, this finding might be attributed to the fact that young children have lower levels of autonomy and mobility than older children and adolescents [120-122]. These lower levels of autonomy and mobility may lead to greater exposure to neighbourhood processes, as these children are more bound to their local neighbourhoods. Previous research also mentioned exposure as a mechanism to explain why social capital had a larger association with the health of parents of young children than adults without young children [123].

For most of the included studies, the investigation of the concept of neighbourhood social capital in relation to social inequalities in health was not the central aim. The few studies that intended to investigate these relations did not integrate their evidence within the specific theoretical [124] and empirical [71] field that links social capital to social inequality in health (i.e., social capital as a mechanism in the relationship between income inequality and health). The scope of this review is new from a theoretical perspective. Analogous to the ways in which psychological and social forces interact in the relationship between income inequality and health [71,124], the present research investigates the workings of social capital in the relationship between individual and neighbourhood socio-economic position and health – the so-called “gradient in health”.

It is clear that separating social capital from a number of related concepts poses a substantial challenge [125]. In the literature, social cohesion has been used to describe communities that are high in social capital [71,97] and collective efficacy [117] and low in social disorganisation.

The quality of the included studies was mostly moderate to strong. Some issues however, prompt caution in the interpretation of the findings. Although six of the studies had a multilevel design, the majority did not adequately explore individual- and neighbourhood-level variance to justify the choice of a multilevel design. Another striking finding was that two of the four studies that did not confirm the current study’s hypotheses utilise a small sample. They sampled between 200 and 475 respondents in 36 to 39 neighbourhoods. Hox [126] provides guidelines concerning sample sizes in multilevel modelling. The 30–30 rule of Kreft states that researchers should strive to sample at least 30 level 2 units (i.e., neighbourhoods, groups, etc.) with at least 30 respondents per level 2 unit. Researchers who are interested in cross-level interactions should aim for an even larger sample size, with at least 50 level 2 units consisting of at least 20 individuals per unit. Consequently, the rather small level 2 sample size may be an important reason why these studies failed to find contextual effects of social capital on the health of children and adolescents.

### Strengths of the study

In this review, a specific focus on studies that measured social capital at the contextual level was pursued. This approach enabled us to maintain a conceptual purity during the review process. Because the purpose of the project ‘Gradient’ is to produce policy guidelines that tackle the social gradient in the health of children and adolescents in local communities, a contextual level approach is the most relevant approach. Furthermore, this review draws attention to the small proportion of social capital research that focusses on children and adolescents and the role of social capital for social inequalities in health.

By pursuing a broad vision on health, the authors also considered studies that focussed on positive health outcomes. Thus, this review complements evidence on the harmful effect of neighbourhoods on health and well-being in children and adolescents with evidence on the promotive effect of social processes in the neighbourhood. The review also considered studies on positive youth development, a topic whose importance is stressed in recent literature [127,128].

### Weaknesses of the study

First, this study was designed to be based on European studies. However, nearly all included studies were conducted in the USA or Canada. Furthermore, several studies focussed on specific populations and minority groups, such as adolescents living in rural areas or African American children, which threatens the generalizability of the results to a more general and European population. Second, a wide range of measures for social capital, SES and health were used. Integration of the evidence is hereby impeded. Third, none of the included studies examined ‘strong’ health indicators (e.g. BMI) or health behaviours; rather, they focussed on outcomes on the border between health and well-being. Finally, this literature review only included evidence from published studies. Consequently, publication bias might have led to an overrepresentation of studies that confirmed hypothesised effects of neighbourhood social capital and, thus, an overestimation of the impact of neighbourhood social capital on children and adolescents.

## Conclusion

### Conclusion and implications for further research

Overall, the results of this review suggest that neighbourhood social capital may partially explain the relationship between socio-economic characteristics and the health of children. Furthermore, the findings suggest that neighbourhood social capital might be particularly beneficial for the health of children in deprived neighbourhoods, although additional research is needed to support this notion.

The findings also illustrate that the beneficial impact of social capital cannot be simplified. Based on the included studies, the factors that contributes to or explain the link between socio-economic factors, neighbourhood social capital and the health of children and adolescents remain unclear. It is possible that only certain characteristics of social capital are significant. The particular conditions that are the most amendable by social capital remain unknown. In addition, one should bear in mind that not all components of social capital are included in this study.

The operationalisation of social capital used in all eight included studies can be situated within theories initially developed by criminologists to explain variations in crime rates across geographic regions. The social disorganisation theory was developed by Chicago school researchers Shaw and McKay [129] and has been linked to the emerging concept of social capital [130]; low stocks of social capital is one of the distinguishing characteristics of socially disorganised communities that are characterised by higher crime rates. In their well-known Chicago study, Sampson et al. [117] developed an index of collective efficacy that combined informal social control with neighbourhood social cohesion and found that collective efficacy was inversely associated with reports of neighbourhood violence, violent victimisation, and homicide rates. All of the included studies used a measure that reflected a form of ‘collective efficacy’, ‘social control’ and ‘social cohesion’ to measure social capital. Most of the included studies used the scale developed by Sampson and colleagues [117] as part of the Project on Human Development in Chicago Neighbourhoods to measure these concepts. However, one can question whether these concepts and this scale are appropriate for research on social capital in a European context, particularly outside of urban areas. After all, social capital is context-dependent. Recent Flemish research found that the scale on informal social control did not sufficiently differentiate Flemish communities [131]. Further research should explore the usefulness of measures of social capital that originate from the Chicago School in a European context and investigate methods to measure social capital that are more closely linked to the theories of the founding fathers of the concept.

To gain insight into the possible value of social capital as a way to tackle the social gradient in health in Europe, research is needed that focusses on European, ‘population-wide’ samples, using data tools appropriate for the European context. When collecting data on minority groups, research should focus on more relevant minority groups in the European context, such as other EU nationals, (descendants from) North-African immigrant workers, and nationals from former European colonies or Roma people. Another important point of interest when measuring social capital is to select indicators and methods that allow interpretation of the direction of associations. Researchers typically utilise the same respondents to report health status and social capital. However, some people tend to provide striking and consistently positive or negative reports when completing a questionnaire, which is known as the negative or positive ‘affect bias’ [132]. Researchers could take this potential source of bias into account in two manners. One method is to use independent samples to report health status and social capital [61]. Another possibility is to use objective measures of social capital to complement the collected data and to ‘correct’ the subjective perception of people’s social capital. This approach was used in one of the included studies [118] .

This literature review suggests that social capital influences the relationship between socio-economic characteristics and health outcomes and well-being in children and adolescents and that social capital might be particularly important for the health of children who reside in deprived neighbourhoods. Although a greater number of studies are needed to provide a more substantive evidence base and better insight into the underlying processes, these findings indicate that social capital might play a role in the health gradient among children and adolescents. Interventions that invest in social capital and target deprived neighbourhoods might help to reach better health for children who reside in low SES neighbourhoods. Furthermore, neighbourhood social capital might foster social processes in the home environment that contribute to good health, such as good parenting practices. Greater insight into the psychosocial resources that are relevant for children who face economic adversity is needed, as the influence of economic hardship on child health contributes to the social gradient in adult health [1,39].

## Competing interest

The authors declare that they have no competing interests.

## Authors’ contributions

VV contributed to the development of the research protocol, screening of studies, completion of the data extraction forms and the quality assessment tools, and wrote the first draft of the paper. BDC contributed to the search strategy, screening of studies, completion of the data extraction forms and the quality assessment tools and finalised the draft of the paper. VS contributed to the research protocol, provided feedback throughout the research process and contributed to the draft of the paper. CCu contributed to the draft of the paper and provided important contributions to the introduction section. CCo, GB, SHJ, SDC and VK contributed to the development of the research protocol, screening of studies and the draft of the paper. LM coordinated the research project and contributed to the development of the research protocol and screening of studies. All authors read and approved the final manuscript.

## Pre-publication history

The pre-publication history for this paper can be accessed here:

http://www.biomedcentral.com/1471-2458/13/65/prepub
